# Transfer learning for data‐efficient abdominal muscle segmentation with convolutional neural networks

**DOI:** 10.1002/mp.15533

**Published:** 2022-02-28

**Authors:** Dónal M. McSweeney, Edward G. Henderson, Marcel van Herk, Jamie Weaver, Paul A. Bromiley, Andrew Green, Alan McWilliam

**Affiliations:** ^1^ Division of Cancer Sciences University of Manchester Manchester UK; ^2^ Radiotherapy Related Research The Christie Foundation Trust Manchester UK; ^3^ Department of Medical Oncology The Christie Hospital NHS Foundation Trust Manchester UK; ^4^ Division of Informatics, Imaging and Data Sciences University of Manchester Manchester UK

**Keywords:** deep learning, sarcopenia, segmentation

## Abstract

**Background:**

Skeletal muscle segmentation is an important procedure for assessing sarcopenia, an emerging imaging biomarker of patient frailty. Data annotation remains the bottleneck for training deep learning auto‐segmentation models.

**Purpose:**

There is a need to define methodologies for applying models to different domains (e.g., anatomical regions or imaging modalities) without dramatically increasing data annotation.

**Methods:**

To address this problem, we empirically evaluate the generalizability of various source tasks for transfer learning: natural image classification, natural image segmentation, unsupervised image reconstruction, and self‐supervised jigsaw solving. Axial CT slices at L3 were extracted from PET‐CT scans for 204 oesophago‐gastric cancer patients and the skeletal muscle manually delineated by an expert. Features were transferred and segmentation models trained on subsets (n=5,10,25,50,75,100,125) of the manually annotated training set. Four‐fold cross‐validation was performed to evaluate model generalizability. Human‐level performance was established by performing an inter‐observer study consisting of ten trained radiographers.

**Results:**

We find that accurate segmentation models can be trained on a fraction of the data required by current approaches. The Dice similarity coefficient and root mean square distance‐to‐agreement were calculated for each prediction and used to assess model performance. Models pre‐trained on a segmentation task and fine‐tuned on 10 images produce delineations that are comparable to those from trained observers and extract reliable measures of muscle health.

**Conclusions:**

Appropriate transfer learning can generate convolutional neural networks for abdominal muscle segmentation that achieve human‐level performance while decreasing the required data by an order of magnitude, compared to previous methods (n=160→10). This work enables the development of future models for assessing skeletal muscle at other anatomical sites where large annotated data sets are scarce and clinical needs are yet to be addressed.

## 1 INTRODUCTION

Segmentation of medical images is a central procedure in extracting imaging biomarkers. In the last decade, assessment of muscle characteristics, by way of muscle segmentation on computed tomography (CT) scans, has enabled further insight into sarcopenia, the degenerative loss of muscle mass and quality associated with aging.^1^ In the context of medical imaging, sarcopenia is assessed via the skeletal muscle index: skeletal muscle area at the L3 vertebral level normalized by patient height.^2^ However, recent studies suggest that skeletal muscle attenuation may be used as an alternative,^3^, ^4^ bypassing the need for patient height, which is often unavailable in anonymized medical data. In oncology, sarcopenia has emerged as an important prognostic factor when treating with chemotherapy,^5^, ^6^, ^7^, ^8^ radiotherapy^9^, ^10^ or surgery,^11^, ^12^, ^13^ where sarcopenia is associated with shorter overall survival and increased toxicity across a variety of disease sites and stages.^14^, ^15^, ^16^

Current methods of sarcopenia assessment are limited as they require time‐consuming manual annotation by a clinician, and automated template‐based approaches, such as the ABACS (*A*utomatic *B* ody *C* omposition *A* nalyzer using *C* omputed tomography image *S* egmentation) module in SliceOMatic (Tomovision) have been shown to perform poorly when the characteristic shape of the muscle compartment is altered, either by anatomical abnormalities or muscle wasting.^17^ Reliance on a statistical shape prior also limits the use of ABACS to CT slices at L3, preventing extraction of skeletal muscle characteristics from scans where the lumbar spine is not imaged (e.g., head and neck cancer radiotherapy planning scans). These limitations have hindered sarcopenia evaluation in clinical practice, especially in radiotherapy patients. Consequently, there has been growing interest in developing fully automated alternatives.

Convolutional neural networks (CNNs) have become the centerpiece of modern segmentation tools.^10^, ^18^, ^19^, ^20^, ^21^ Such models lead to impressive results whilst limiting human intervention (following annotation of training data) by removing the need for feature engineering. Features are learned entirely via the optimization process. Although this facilitates model development, the result is a black‐box that requires large amounts of training data to ensure generalizability, from which insight is hard to gain. In the context of skeletal muscle segmentation, Park et al.^18^ developed a fully convolutional network (FCN) trained on 883 CT scans. Weston et al.^20^ and Edwards et al. ^21^ trained a U‐Net^22^ with 2430 and 682 images, respectively. The quantity of training data required prohibits wide application of these methods. As skeletal muscle delineations are not a routine by‐product of treatment planning, large annotated data sets are time‐consuming to curate and become a limiting factor for models that need to be retrained on different cohorts or anatomical sites.

A number of approaches have been taken to allow CNN training on limited data. These fall under two categories: augmenting the data set or altering the training dynamics. The former involves generating synthetic data by randomly applying transformations (e.g. rotations, elastic deformations, and random erasing) under the assumption that more information can be extracted from the augmented data set.^23^ The latter encompasses a number of techniques that seek to modify the network architecture or the learning procedure to enable improved performance on small data sets.

Transfer learning alters the training dynamics by using a sequence of tasks to produce a final model. In network‐based transfer learning,^24^ an initial network is trained on a pretext task with large amounts of data. The learned features are then transferred to a target model, where they serve to initialize network layers, for training on a target task where few annotated data are available. The central assumption being that the features learned on the pretext task are also useful for the target task. Indeed, it has been shown that early layers learn low‐level features such as color blobs and Gabor filters, regardless of the data set or training objective.^25^, ^26^ This dictionary of fundamental features can therefore be used across domains and tasks.

To the best of our knowledge, only two publications have applied transfer learning to skeletal muscle segmentation. Lee et al.^19^ and Green et al.^10^ both used a VGG‐16^27^ pre‐trained on ImageNet, a large‐scale natural image data set comprising over 14 million images belonging to 1000 categories,^28^ to initialize the encoder in an FCN or U‐Net architecture, respectively. Although the authors train their models on much smaller data sets (250 and 160 axial CT slices, respectively) compared to those discussed previously,^18^, ^20^, ^21^ we believe that further research surrounding optimization of the transfer learning procedure will allow much smaller training set sizes and in consequence, more adaptable models.

We seek to decrease the time and cost required to develop accurate muscle segmentation models, to facilitate adaptation to different anatomical regions or patient cohorts. To this end, we investigate optimal transfer learning practices in segmenting medical images via the use case of skeletal muscle delineation at the L3 vertebral level. We examine the relationship between training set size and segmentation accuracy for a number of pretext tasks. Our results are compared to our baseline, an inter‐observer study performed by 10 trained radiographers. Finally, we compare measures of muscle area and attenuation with those from our gold‐standard delineations to emphasize the clinical viability of our method.

## 2 METHODOLOGY

### 2.1 Data Preparation

The analysis was performed on an oesophago‐gastric cancer cohort (n=204) where single PET‐CT slices were manually extracted at the L3 vertebral level. Delineations of skeletal muscle were completed by a clinical expert (**JW**) with 6 years of expertise. A hold‐out test set (n=37) was formed and annotated by trained observers (see 2.4). The remaining data (n=167) were separated into four folds.

**FIGURE 1:**
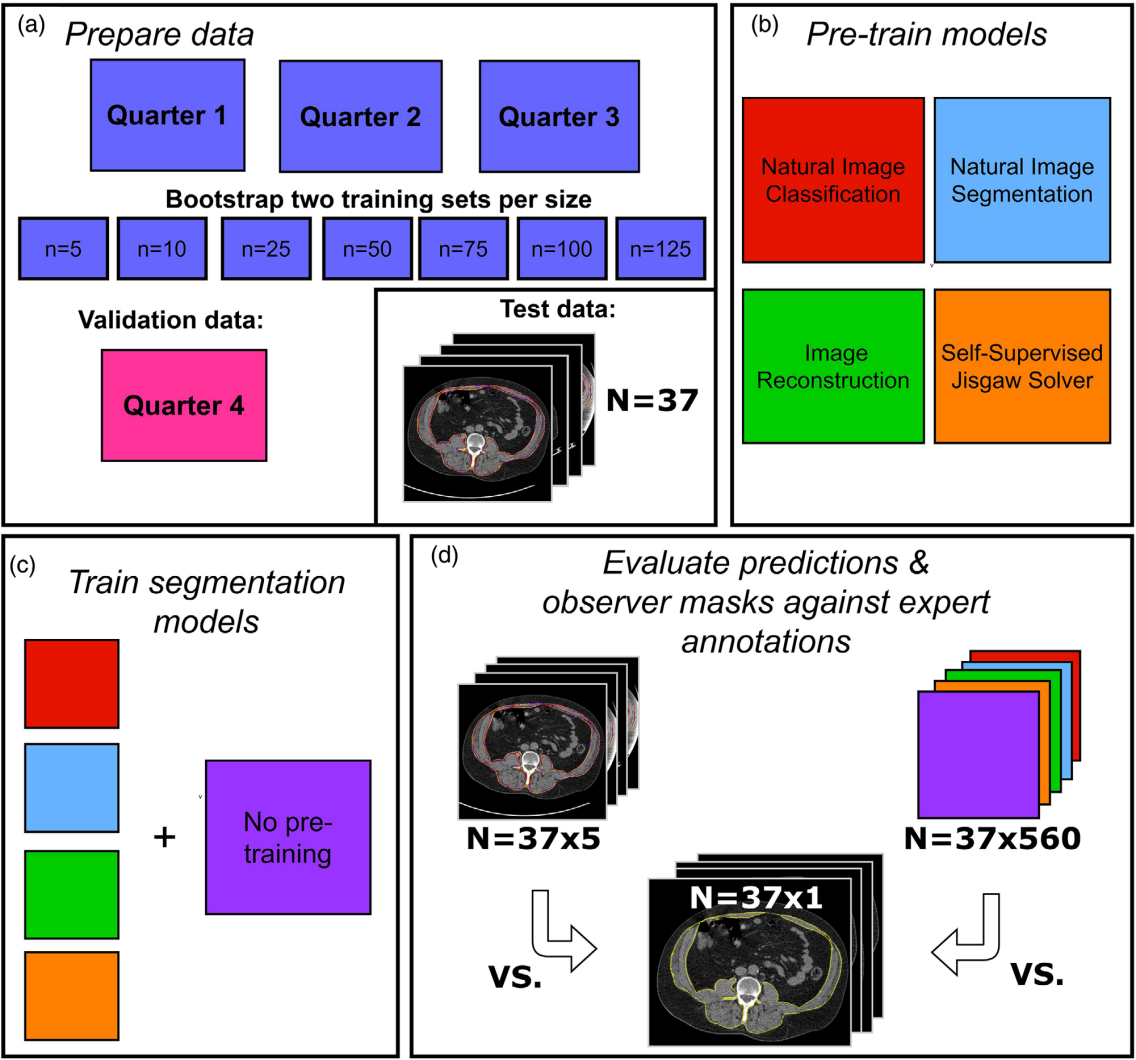
Experimental workflow. Fourfold cross‐validation was performed. (a) Training sets of varying sizes were sub‐sampled from the resulting training & validation split. During training, the excluded fold was used as validation data. Images used for our observer study were kept independent and formed the test set. (b) Four pretext tasks were used for pre‐training: image classification, image segmentation, unsupervised image reconstruction, and self‐supervised jigsaws. (c) Weights from pre‐trained models were transferred to a FCN‐ResNet101 trained to segment skeletal muscle on each subset until convergence. Randomly initialized models were also trained on each subset for comparison. (d) Model predictions and observer masks were evaluated on the same test set (n=37) with expert gold‐standard delineations

During training, one of the folds was used for validation while the remaining folds were combined to form a parent training set. The parent training set was randomly subsampled to produce independent training subsets of varying sizes (n=5,10,25,50,75,100,125). Larger subsets were generated by incrementally expanding the smaller subsets. For example, subsets with ten images were produced by adding five new samples to the existing subset of five images. We elected to generate two subsampled data sets per size. For each subset, two models were trained to account for the stochasticity of the optimization/initialization procedure. An illustration of the experimental workflow is shown in Figure 1.

### 2.2 Pre‐Training

We tested four pre‐training methods:

(1) Image classification on natural images.
(2) Image segmentation on natural images.
(3) Unsupervised image reconstruction of the training data.
(4) Self‐supervised approach using jigsaw (puzzle) solving on the training data.

A number of large, publicly available data sets are commonly used for developing and testing image classification models. ImageNet is one such data set consisting of over 14 million natural images belonging to 1000 different classes.^28^ In this work, we used a ResNet101 pre‐trained on ImageNet, available through the PyTorch framework^1^.

In PyTorch, image segmentation models are pre‐trained on Microsoft's Common Objects in Context (COCO) natural image data set, widely used for object detection, image captioning, and semantic image segmentation.^29^ We opted to use the fully convolutional ResNet101 (FCN‐ResNet101^2^) due to its similarity to the classification model pre‐trained on ImageNet.

Convolutional autoencoders were used for unsupervised image reconstruction, a task with the aim of extracting high‐level features from an input CT slice and reconstructing the initial image from the extracted features. We trained a randomly initialised FCN‐ResNet101 to reconstruct the CT slices in the original training set (N=167) by minimizing the mean‐squared error between the input image and the reconstructed slice. As the target network expects three channel inputs, we converted the initial single‐channel image by copying the input across three‐channels and applied channel‐wise normalization according to the ImageNet mean and standard deviation for each channel.

Finally, a self‐supervised approach to solving jigsaw puzzles was used to extract features from CT slices.^30^ We use the full training set (N=167) for training. Input slices were divided into 3x3 grids from which nine patches (per image) were extracted. A set of all possible permutations was filtered such that the top 50 with the greatest Hamming distance were used. The Hamming distance is defined as the number of differing elements between sets. In other words, we selected the top 50 most different permutations of the input patches. The patches were then shuffled according to one of these permutations and a Siamese CNN (nine ResNet101 CNNs with shared weights) was then trained to predict the input permutation by minimizing the cross‐entropy loss across all predictions. As with the auto‐encoder, we converted the single‐channel images to three‐channel CT slices and applied channel‐wise normalization. Training and validation curves for auto‐encoder and jigsaw pre‐training are presented in Appendix A.

### 2.3 Experiments

Due to the nature of the aforementioned tasks, different CNN model architectures were used. To account for this, weights from layers of the pre‐trained models were directly transferred to the target network (FCN‐ResNet101) if they were also present in the latter. Consequently, layers of the target model that did not appear in the pre‐trained architecture were randomly initialized.

Target models were independently trained on each training set by minimizing a combined binary cross‐entropy ($L_{BCE}$) and Dice loss ($L_{DSC}$),^31^, ^32^ until both the training and validation losses had saturated. Loss functions were defined as follows:

(1) $$\begin{array}{ccc} L_{\mathrm{BCE}} & = & -\frac{1}{\mathrm{HW}}\sum_{i,j}\left[Y_{ij}\cdot \log\left({\hat{Y}}_{ij}\right)+\left(1-Y_{ij}\right)\right.\\ & & \left.\cdot \log\left(1-{\hat{Y}}_{ij}\right)\right],0\leq i,j\leq H,W \end{array}$$

(2) $$\begin{array}{c} L_{\mathrm{DSC}}=1-\frac{2|\hat{Y}\cap Y|}{|\hat{Y}\left|+|Y\right|} \end{array}$$

(3) $$\mathrm{Loss}=\frac{1}{N}\sum_{n}^{N}\left({L_{\mathrm{BCE}}}^{\left(n\right)}+{L_{\mathrm{DSC}}}^{\left(n\right)}\right)$$

where H,W are the height and width of the input image and N is the number of samples in a batch. The gold‐standard and predicted masks are denoted Y and $\hat{Y}$, respectively (where $Y,\hat{Y}\in R^{H\times W}$). Training and validation curves can be found in Appendix B.

The Adam optimizer^33^ was used for optimization with an initial learning rate of $3\times 10^{-3}$ in models without pre‐training. This was decreased to $3\times 10^{-4}$ when transferring weights from a previously trained model, to fine‐tune the learned features. The training procedure was repeated to account for stochastic optimization and weight initialization in randomly initialized layers.

Data augmentation was applied in an identical manner for all models and consisted of randomly applying a combination of horizontal flipping, rotations ($\pm 20^{\circ }$), elastic deformations, and scaling. Inputs were clipped according to a window and level of 400 Hounsfield units (HU) and 50 HU, respectively. Single‐channel CT slices were converted to three‐channel images by repeating the image three times along the channel axis (as expected by the FCN‐ResNet101 architecture) and each channel was then normalized according to the ImageNet mean and standard deviation for that channel. Model weights were saved at the minimum of the validation loss and were subsequently used for analysis.

Our analysis consisted of performing a single forward pass of the test data through each model and recording the predictions. The result is a single‐channel image of the per‐voxel class predictions (foreground or background). Finally, a sigmoid function was applied for conversion to a binary mask (a process which was handled by the loss function at training).

To quantify the accuracy of the output segmentations, the Dice similarity coefficient (DSC)^32^ and root mean square distance‐to‐agreement (RMS‐DTA) between each prediction and its corresponding gold‐standard annotation were calculated. The former provides a measure of overlap between predicted masks and the gold‐standard, while the latter is a distance metric between predicted and gold‐standard boundaries. They are defined as follows:

(4) $$\begin{array}{ccc} \text{DSC} & = & \frac{2|\hat{Y}\cap Y|}{|\hat{Y}\left|+|Y\right|}\\ \text{RMS-DTA} & = & \sqrt{\frac{1}{K}\sum_{k}^{K}{\left[d\left({\hat{Y}}_{k},Y_{k}\right)-d\left(Y_{k},{\hat{Y}}_{k}\right)\right]}^{2}} \end{array}$$

where K is the total number of points on the predicted boundary and d is the signed distance from a point on the predicted boundary (${\hat{Y}}_{k}$) and the nearest point on the reference boundary ($Y_{k}$).

Finally, we compared extracted measures of muscle quality, skeletal muscle density (mean HU within the mask, SMD), and skeletal muscle area (Number of foreground pixels × pixel area (in cm^2^), SMA), with those from our gold‐standard delineations. To mitigate the impact of partial volume effect on measures of muscle density, post‐processing was applied to model predictions. This consisted in applying a threshold of 175 HU to the original CT volumes to produce a binary mask of high density regions such as bones. The mask was then isotropically expanded by 2 mm. Bone masks were then removed from model predictions. This served to diminish the effect of neighboring bony anatomy on extracted measures of muscle density.

### 2.4 Inter‐observer variation

**FIGURE 2:**
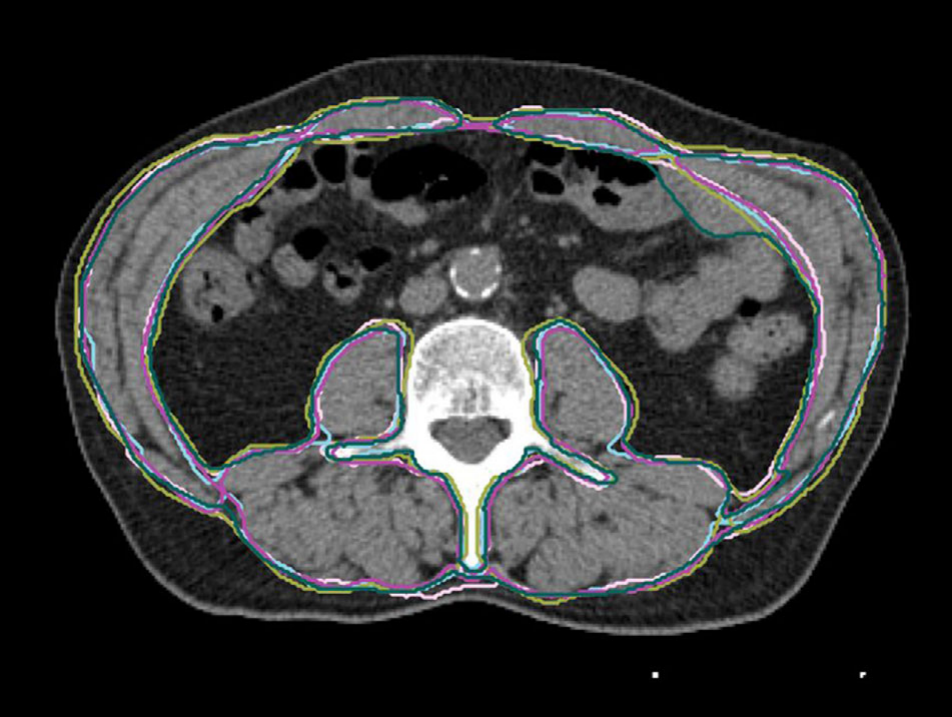
An example CT slice from our inter‐observer study, with multiple observer delineations in different colors

To establish a reliable estimate of human‐level performance, we investigated inter‐observer variation. Ten radiographers were given access to an in‐house segmentation tool (**MvH**). Although the observers had expertise in analyzing routine medical images (Median = 10 years, Range = 3–25 years), they had no prior training for the task. Initially, the participants undertook a training protocol consisting of contouring skeletal muscle in three training images (CT slices at L3). Subsequently, feedback was provided by a clinical expert (**JW**), the radiographers were split into two groups, and were each assigned 20 images from the test set. As a result, six segmentations (five from observers and one expert) were available for each test image—three images only had four observer segmentations due to technical difficulties (Figure 2).

Observer variability was then quantified by calculating mean DSC and mean RMS‐DTA for every image, providing a target for model performance. Finally, to determine the role of observer variation on muscle characteristics (SMD, SMA), we calculated the mean difference against our expert contours.

Dunnett's tests were performed to identify significant differences in segmentation accuracy (DSC & RMS‐DTA) between model predictions and observer delineations; and were used to identify significant differences in extracted muscle characteristics (SMD & SMA) between expert delineations and model predictions.

## 3 RESULTS

From our observer study, we determined that trained observers achieved a mean DSC of 0.901±0.003 and a mean RMS‐DTA of 0.318±0.029 cm, when evaluated against expert delineations. These measures of segmentation accuracy were associated with the following mean differences (observer‐expert) in extracted muscle characteristics: SMD Variability = −6.045±5.529 HU and SMA Variability = −5.135±10.303 cm^2^.

**FIGURE 3:**
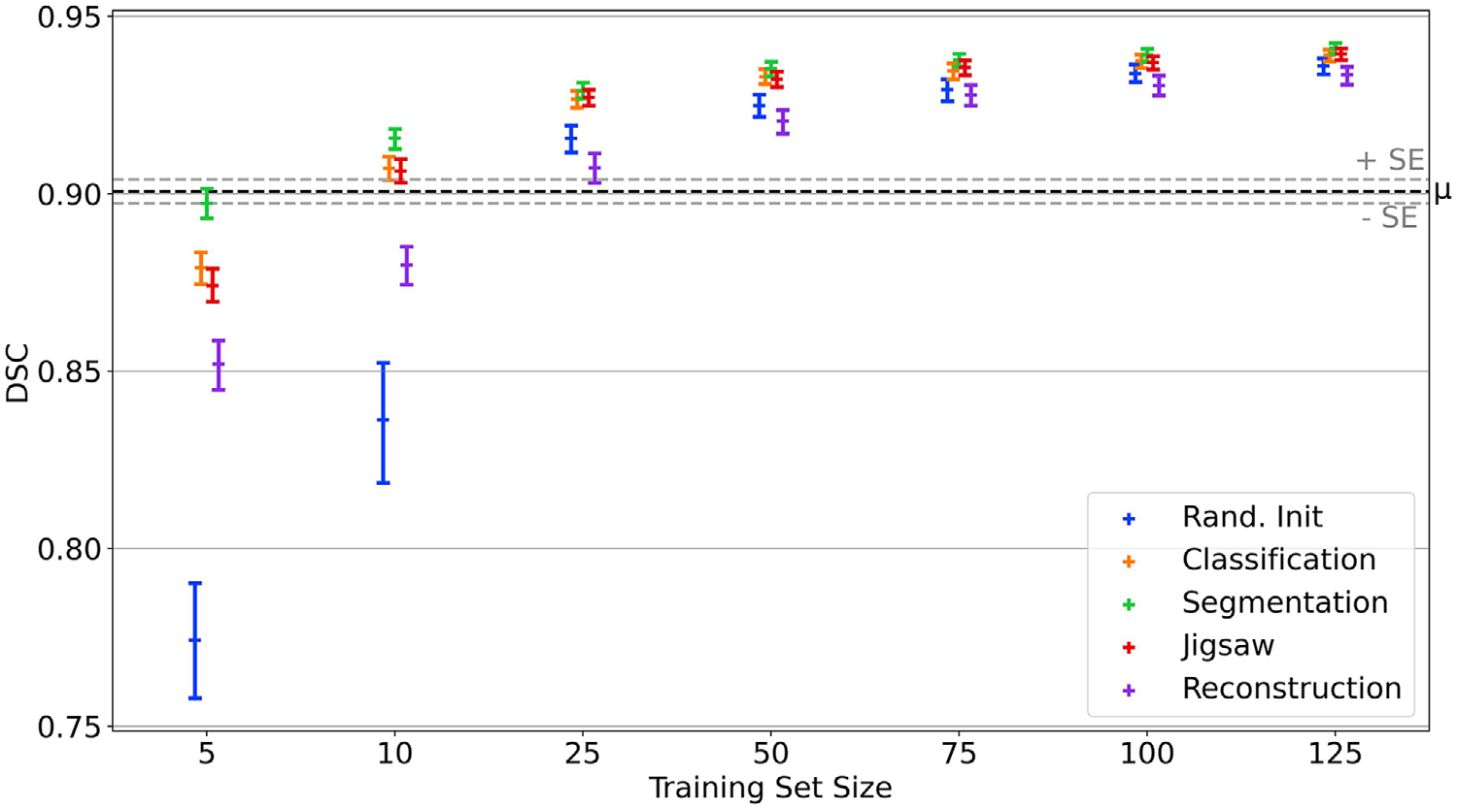
Mean DSC, of CNN predictions evaluated against expert gold‐standard, as a function of training set size. Error bars represent 95% confidence intervals. Mean observer variation (μ; ± standard error (SE)) was found by calculating mean DSC for all observer segmentations against the clinical expert's

**FIGURE 4:**
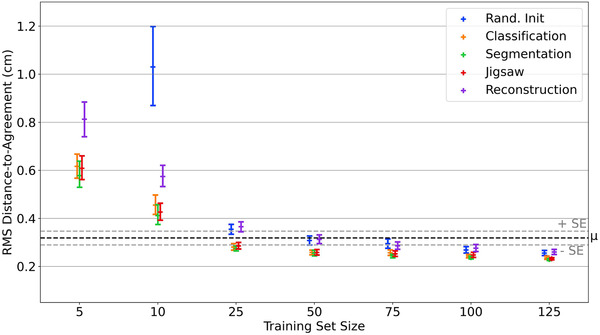
Mean RMS‐DTA (in cm), of CNN predictions evaluated against expert gold‐standard, as a function of training set size. Error bars represent 95% confidence intervals. Note, results from the sixteen randomly initialized models at n=5 have been omitted as the mean RMS‐DTA was 1.72±0.10 cm. Mean observer variation (μ; ± standard error (SE)) was found by calculating mean RMS‐DTA for all observer segmentations against the clinical expert's

DSC, between CNN predictions and expert gold‐standard, as a function of training set size are shown in Figure 3 where different colors represent different pretext tasks. RMS‐DTA results are displayed in Figure 4. In these figures, each point represents the mean and 95% confidence interval for all predictions from 16 models. Dotted lines indicate mean score and the associated standard error for all observers across the test set.

From Figure 3 and Table 1, all models trained on n≥50 patients produce segmentations with a DSC that is better than trained observers, regardless of pre‐training strategy (p<0.001). In the case of RMS‐DTA, all models trained on n≥25 produce delineations that are not significantly different to those manually generated by observers (p>0.001, see Table 1 and Figure 4). As training set sizes increase, differences between pretext tasks decrease: all models converge toward a common value (DSC ≈0.94). Regardless of source task, model performance plateaus as n exceeds 100 (see Appendix D). Beyond n≥25, there is no significant difference between models pre‐trained on image segmentation and those pre‐trained on image classification or jigsaw solving (see Appendix C).

**TABLE 1:** Resulting *p*‐values from performing Dunnett's tests to identify significant differences in DSC (*Top*) & RMS‐DTA (*Bottom*) between model predictions and observer delineations (control=observer delineations). Models that outperformed observers are indicated in bold and models that were significantly worse are underlined

*Source Task*	n=5	n=10	n=25	n=50	n=75	n=100	n=125
Rand. Init.	$\underline{p<0.001}$	$\underline{p<0.001}$	0.038	p<0.001	p<0.001	p<0.001	p<0.001
Classification	$\underline{p<0.001}$	0.777	p<0.001	p<0.001	p<0.001	p<0.001	p<0.001
Segmentation	0.999	0.036	p<0.001	p<0.001	p<0.001	p<0.001	p<0.001
Jigsaw	$\underline{p<0.001}$	0.885	p<0.001	p<0.001	p<0.001	p<0.001	p<0.001
Reconstruction	$\underline{p<0.001}$	$\underline{p<0.001}$	0.746	p<0.001	p<0.001	p<0.001	p<0.001

Rand. Init.	$\underline{p<0.001}$	$\underline{p<0.001}$	0.999	1.000	1.000	0.971	0.833
Classification	$\underline{p<0.001}$	0.077	0.999	0.864	0.785	0.714	0.482
Segmentation	$\underline{p<0.001}$	0.395	0.992	0.826	0.644	0.550	0.440
Jigsaw	$\underline{p<0.001}$	0.245	0.999	0.864	0.785	0.714	0.482
Reconstruction	$\underline{p<0.001}$	$\underline{p<0.001}$	0.983	1.000	1.000	0.996	0.892

At the smallest training set sizes (n=5,10), the choice of pretext task becomes more important. Natural image segmentation is the optimal choice. At n=5, this is the only approach that leads to DSC scores that are not significantly different to trained observers. At training set size n=10, pre‐training on image segmentation, image classification or jigsaw solving all lead to models that are not significantly different to trained observers, both in terms of DSC and RMS‐DTA (Table 1). Nevertheless, image segmentation still outperforms the other methods at n=10 (see Figures 3 and 4; Appendix C). In addition to improved performance, increasing training set size leads to improved model generalizability as highlighted by narrowing confidence intervals in Figures 3 and 4.

**FIGURE 5:**
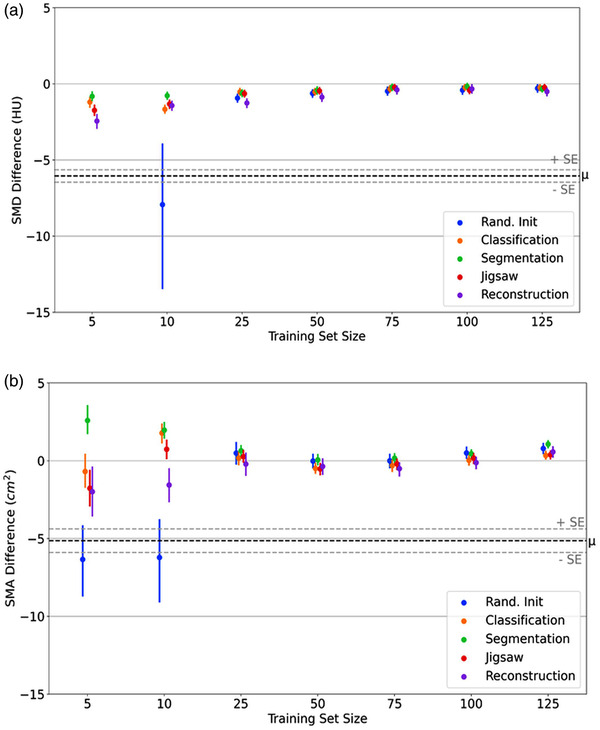
(a) Difference in skeletal muscle density extracted from model predictions and expert gold‐standard. Defined as prediction—gold‐standard. Note that results from the 16 randomly initialized models at n=5 have been omitted as the mean difference was −47.02±10.48 HU. (b) Difference in skeletal muscle area between predictions and gold‐standard, defined as above. Dotted lines indicate mean observer difference and the associated standard error (μ±SE)

Differences in muscle features (SMD & SMA) between CNN predictions and expert gold‐standard values are displayed in Figure 5. As expected, as n increases, the values extracted from our models approach those of our gold‐standard delineations. When comparing segmentation metrics, the choice of source task plays an important role, especially at the smallest training set sizes. When comparing extracted measures of muscle quality, however, the choice of source task is not as important. Table 2 shows that all models can extract muscle characteristics comparable to our gold‐standard, with the exception of SMD for the set of randomly initialized models trained on n=5. Note, that these models have been omitted from Figure 5 (*top*) as the mean difference was −47.02±10.48 HU. These results support our hypothesis that accurate and clinically useful segmentation models can be trained on much smaller data sets than currently used.

**TABLE 2:** Resulting *p*‐values from performing Dunnett's tests to identify significant differences in SMD (*Top*) & SMA (*Bottom*) between model predictions and expert delineations (control=expert delineations). Models that extracted significantly less accurate muscle characteristics are underlined

*Source Task*	n=5	n=10	n=25	n=50	n=75	n=100	n=125
Rand. Init.	$\underline{p<0.001}$	0.306	1.0	1.0	1.0	1.0	1.0
Classification	1.0	1.0	1.0	1.0	1.0	1.0	1.0
Segmentation	1.0	1.0	1.0	1.0	1.0	1.0	1.0
Jigsaw	1.0	1.0	1.0	1.0	1.0	1.0	1.0
Reconstruction	1.0	1.0	1.0	1.0	1.0	1.0	1.0

Rand. Init.	0.641	0.655	1.0	1.0	1.0	1.0	1.0
Classification	1.0	1.0	1.0	1.0	1.0	1.0	1.0
Segmentation	0.999	1.0	1.0	1.0	1.0	1.0	1.0
Jigsaw	1.0	1.0	1.0	1.0	1.0	1.0	1.0
Reconstruction	1.0	1.0	1.0	1.0	1.0	1.0	1.0

## 4 DISCUSSION

We present an investigation into the optimal methodology for developing data‐efficient skeletal muscle segmentation models. We compare four pretext tasks: image classification, semantic image segmentation, unsupervised image reconstruction, and a self‐supervised approach to solving jigsaws. We transfer learned weights to target segmentation models, which we then optimize on training sets of varying sizes and compare to randomly initialized models. Human‐level performance was established via an inter‐observer study consisting of ten radiographers and acted as a baseline against which models were compared.

To the best of our knowledge, this work is the first to empirically evaluate the generalizability of models pre‐trained on different tasks, where the target task is medical image segmentation. Typically, image classification on ImageNet is used as a pretext task.^34^ Our results suggest that in the domain where n≥50, all models converge and significantly outperform trained observers as measured by DSC (see Table 1 and Figure 3). In terms of RMS‐DTA, Table 1 and Figure 4 show that models trained on n≥25 lead to predictions that are not significantly different to trained observers, independent of source task. In this domain, there are no significant differences between models pre‐trained on image segmentation, image classification, and jigsaw solving (see Appendix C). As n increases beyond n=100, model performance begins to plateau, irrespective of source task (p>0.001, Appendix D). We also note that as training set size increases, variability in segmentation accuracy decreases, probably highlighting a decrease in fit failures.

At the smallest training set sizes (n=5,10), models pre‐trained on image segmentation outperform other methods (see Figures 3 and 4; Appendix C) and lead to predictions that are not significantly different to observers (see Table 1). The choice of pretext task is most important when very few samples (n<25) are available for fine‐tuning. We find that all models can extract muscle characteristics comparable to those from our expert delineations (see Table 2), except randomly initialized models at n=5.

Our results are limited in that we investigate model performance on one target task and domain, namely skeletal muscle segmentation at L3 on axial PET‐CT slices. Future work will seek to validate our results across vertebral levels and imaging modalities. It should be noted that data augmentation played an essential role in preventing overfitting and may be responsible for the good performance of our models at small training set sizes. As such, transfer learning is not solely responsible. Nevertheless, data augmentation techniques are widely available and easily integrable into any segmentation pipeline. As a single expert was available for data annotation, we have assumed that their gold‐standard annotations are optimal. It may be interesting to investigate how performance is affected when training on multiple expert annotations, removing potential bias introduced by using a single observer. Similarly, the relatively large variability at the smallest training set sizes (n=5,10) could be related to randomly sampling delineations of different quality. It may be of interest to determine if results can be improved by initially screening the parent data set and removing lower quality annotations.

We build models trained on as few as 10 patients that achieve human‐level segmentation accuracy and extract measures of muscle quality that are not significantly different to those from our expert gold‐standard. Our approach thus reduces the cost and time needed to curate training sets for skeletal muscle segmentation models. As a consequence, this facilitates extension of these tools to other anatomical regions, where the necessity for large data sets make current approaches unfeasible. The ability to easily and cheaply adapt muscle segmentation models to a variety of sites will enable the integration of sarcopenia evaluation into routine care and allow large‐scale retrospective analyses of (currently) under‐studied patient groups.

## 5 CONCLUSION

We show that transfer learning, used precisely, can be leveraged to produce data‐efficient skeletal muscle segmentation models—decreasing the required data by an order of magnitude compared to previous methods. Importantly, this enables extension of such models to anatomical sites where large annotated data are scarce but clinical needs are still unmet.

We find that models pre‐trained on an image segmentation task and fine‐tuned on 10 patients lead to measures of segmentation accuracy comparable to our trained observers. They also extract measures of muscle health comparable to those extracted by expert, manual delineations.

## CONFLICT OF INTEREST

The authors have no conflict to disclose.

## Figures and Tables

**FIGURE A1:**
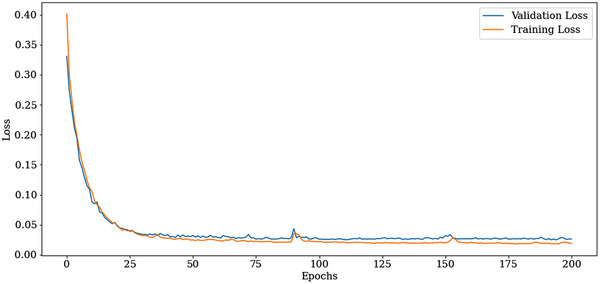
Training and validation loss curves for autoencoder pre‐training, demonstrating that the source model was trained to convergence

**FIGURE A2:**
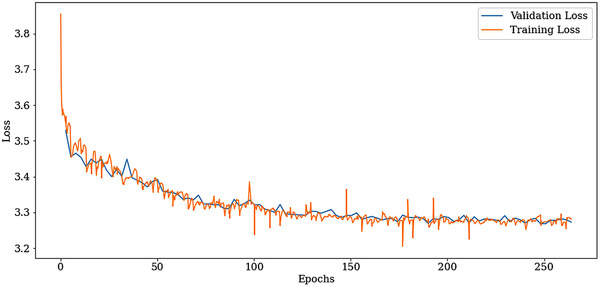
Training and validation loss curves for jigsaw solving pretext task, demonstrating that the source model was trained to convergence

**FIGURE B1:**
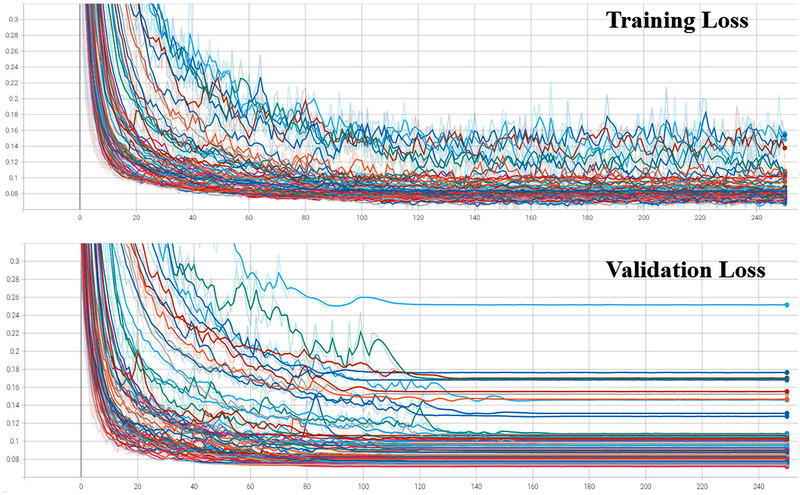
Training and validation losses versus epochs for 50 randomly sampled models

## Footnotes

1
https://pytorch.org/docs/stable/torchvision/models.html#torchvision.models.resnet101

2
https://pytorch.org/docs/stable/torchvision/models.html#torchvision.models.segmentation.fcn_resnet101

## APPENDIX A PRE‐TRAINING CURVES

### A.1 Unsupervised Image Reconstruction—Convolutional Autoencoder

Training and validation loss for unsupervised image reconstruction pretext task (Figure A1).

### A.2 Self‐Supervised Jigsaw Solving

Training and validation loss for self‐supervised jigsaw solving pretext task (Figure A2).

## APPENDIX B REPRESENTATIVE TRAINING CURVES

Training and validation curves used to monitor model training, described in Section 2.3. Combined loss (Dice & binary cross‐entropy loss) on the y‐axis and training epoch on the x‐axis. We have presented a random subset (n=50) of all trained models (n=560) to provide a clearer visualization (Figure B1).

## APPENDIX C PER TRAINING SET SIZE—INTER‐MODEL MANN–WHITNEY U‐TEST

Inter‐model Mann–Whitney U‐tests were performed at every training set size, resulting *p*‐values are presented below. RMS‐DTA was used as metric as it is a more robust estimate of segmentation accuracy compared to DSC. ∗∗∗∗ indicates significance at p=0.001

### C.1 *N*=5

**Table:**

	Jigsaw	Classification	Rand. Init.	Segmentation	Reconstruction
Jigsaw	1.0	0.2694	****	****	****
Classification	0.2694	1.0	****	****	****
Rand. Init.	****	****	1.0	****	****
Segmentation	****	****	****	1.0	****
Reconstruction	****	****	****	****	1.0

### C.2 *N*=10

**Table:**

	Jigsaw	Classification	Rand. Init.	Segmentation	Reconstruction
Jigsaw	1.0	0.5638	*****	****	****
Classification	0.5638	1.0	****	****	****
Rand. Init.	****	****	1.0	****	0.9588
Segmentation	****	****	****	1.0	****
Reconstruction	****	****	0.9588	****	1.0

### C.3 *N*=25

**Table:**

	Jigsaw	Classification	Rand. Init.	Segmentation	Reconstruction
Jigsaw	1.0	0.4806	***	0.9723	***
Classification	0.4806	1.0	***	0.4553	***
Rand. Init.	***	***	1.0	***	***
Segmentation	0.9723	0.4553	***	1.0	***
Reconstruction	***	***	***	***	1.0

### C.4 *N*=50

**Table:**

	Jigsaw	Classification	Rand. Init.	Segmentation	Reconstruction
Jigsaw	1.0	0.7182	***	0.1848	***
Classification	0.7182	1.0	***	0.3653	***
Rand. Init.	***	***	1.0	***	***
Segmentation	0.1848	0.3653	***	1.0	***
Reconstruction	***	***	***	***	1.0

### C.5 *N*=75

**Table:**

	Jigsaw	Classification	Rand. Init.	Segmentation	Reconstruction
Jigsaw	1.0	0.5641	****	0.9997	****
Classification	0.5641	1.0	****	0.5645	****
Rand. Init.	****	****	1.0	****	0.1332
Segmentation	0.9997	0.5645	****	1.0	****
Reconstruction	****	****	0.1332	****	1.0

### C.6 *N*=100

**Table:**

	Jigsaw	Classification	Rand. Init.	Segmentation	Reconstruction
Jigsaw	1.0	0.7497	0.0521	0.1863	****
Classification	0.7497	1.0	0.0184	0.3398	****
Rand. Init.	0.0521	0.0184	1.0	****	0.0100
Segmentation	0.1863	0.3398	****	1.0	****
Reconstruction	****	****	0.0100	****	1.0

### C.7 *N*=125

**Table:**

	Jigsaw	Classification	Rand. Init.	Segmentation	Reconstruction
Jigsaw	1.0	0.7402	0.0191	0.4232	****
Classification	0.7402	1.0	0.0392	0.2392	****
Rand. Init.	0.0191	0.0392	1.0	0.0012	0.0958
Segmentation	0.4232	0.2392	****	1.0	****
Reconstruction	****	****	0.0958	****	1.0

## APPENDIX D INTRA‐MODEL MANN–WHITNEY U‐TEST

Intra‐model Mann–Whitney U‐tests were performed at every training set size less than 125 using the equivalent model at n=125 as the other sample. Resulting *p*‐values are presented below. RMS‐DTA was used as metric as it is a more robust estimate of segmentation accuracy compared to DSC. ∗∗∗∗ indicates significance at p=0.001.

**Table:**

	n=5	n=10	n=25	n=50	n=75	n=100
Jigsaw	****	****	****	****	0.0234	0.0805
Classification	****	****	****	****	0.0111	0.2547
Rand. Init.	****	****	****	****	****	0.2187
Segmentation	****	****	****	****	****	0.1606
Reconstruction	****	****	****	****	****	0.0312
